# Supplementary material for: Alterations in Gene Expression and the Fatty Acid Profile Impact but Do Not Compromise the In Vitro Maturation of Zebrafish (Danio rerio) Stage III Ovarian Follicles after Cryopreservation
Source: Animals (Basel). 2023 Nov 18;13(22):3563. doi: 10.3390/ani13223563 (PMC10668701; doi:10.3390/ani13223563)
Supplement: Supplementary file 1 [file animals-13-03563-s001.zip › animals-2602207-supplementary.pdf]

Table S1: Fatty acid profile of the neutral fraction (triglycerides - TG) of stage III ovarian follicles of zebrafish (*Danio rerio*).

Fatty Acids	Treatments		*p* value
	Control	Vitrified	
12:0	12.64 ± 6.85	ND	-
13:0	1.37 ± 0.17	ND	-
14:0	1.39 ± 0.34	1.25 ± 0.48	0.823
15:0	1.86 ± 0.18	ND	-
16:0	27.16 ± 2.68	32.18 ± 0.18	0.114
16:1n7	2.35 ± 0.23	3.19 ± 0.59	0.254
17:0	2.79 ± 0.57	ND	-
17:1	0.81 ± 0.11	ND	-
18:0	11.01 ± 1.14	8.61 ± 0.30	0.111
18:1n9	23.91 ± 1.65	36.30 ± 0.67	0.002
18:1n7	2.92 ± 0.36	2.55 ± 0.32	0.489
18:2n6	5.47 ± 0.81	3.77 ± 0.39	0.131
18:4n3	0.99 ± 0.16	ND	-
20:1n9	ND	1.22 ± 0.69	-
20:5n3	0.88 ± 0.09	1.20 ± 0.23	0.270
20:2n6	ND	2.31 ± 0.24	-
20:3n6	0.89 ± 0.11	0.79 ± 0.05	0.453
20:4n6	1.12 ± 0.21	2.05 ± 0.21	0.034
21:0	0.60 ± 0.12	ND	-
22:1	ND	0.81 ± 0.55	-
22:2n6	ND	0.96 ± 0.29	-
22:4n6	ND	0.58 ± 0.02	-
22:5n3	ND	0.97 ± 0.41	-
22:6n3	0.59 ± 0.02	0.87 ± 0.00	0.000
24:1	1.26 ± 0.27	ND	-
∑SFA	58.82 ± 3.40	42.43 ± 0.35	0.008
∑MUFA	31.25 ± 2.37	44.07 ± 1.49	0.010
∑PUFA	6.36 ± 0.92	7.83 ± 0.98	0.333
∑LCPUFA	3.58 ± 0.36	5.67 ± 0.86	0.089
∑PUFA n3	2.46 ± 0.18	3.04 ± 0.63	0.433
∑PUFA n6	7.48 ± 0.90	10.46 ± 1.21	0.119
Ratio n3/n6	0.33 ± 0.02	0.28 ± 0.03	0.233
Ratio DHA/ARA	0.57 ± 0.11	0.43 ± 0.05	0.335
Ratio ARA/EPA	1.30 ± 0.30	1.79 ± 0.21	0.248
Ratio DHA/EPA	0.69 ± 0.08	0.80 ± 0.19	0.598

Values represent means ± standard error of the mean (n = 3 animals per treatment). [a,b] Different letters indicate statistical differences between the experimental diets by Tukey's test ($p < 0.05$), the letter a refers to the highest value while the letter b refers to the lowest. ∑SFA, ∑MUFA, ∑PUFA, ∑LC-PUFA, ∑n-3 PUFA, ∑n-6 PUFA are the sum of saturated fatty acids (SFA), monounsaturated (MUFA), polyunsaturated (PUFA), polyunsaturated fatty acids from long chain (LC-PUFA), n3 polyunsaturated (n-3 PUFA) and n6 polyunsaturated (n-6 PUFA), respectively. DHA/ARA is the ratio between the sum of total DHA

(docosahexaenoic acid, 22:6n-3) and ARA (arachidonic acid, 20:4n-6); ARA/EPA is the ratio between the sum of total ARA (arachidonic acid, 20:4n-6) and EPA (eicosapentaenoic acid, 20:5n-3); and DHA/EPA is the ratio between the sum of total DHA (docosahexaenoic acid, 22:6n-3) and EPA (eicosapentaenoic acid, 20:5n-3).

Table S2: Fatty acid profile of the polar fraction (phospholipids - PL) of stage III ovarian follicles of zebrafish (*Danio rerio*).

Fatty Acids	Treatments		*p* value
	Control	Vitrified	
12:0	7.30 ± 1.23	ND	-
13:0	1.25 ± 0.12	ND	-
14:0	1.16 ± 0.13	0.40 ± 0.16	0.021
15:0	1.57 ± 0.24	ND	-
15:1	ND	1.79 ± 0.01	-
16:0iso	1.40 ± 0.02	ND	-
16:0	27.30 ± 1.36	24.79 ± 0.19	0.141
16:1n7	0.49 ± 0.08	0.44 ± 0.13	0.765
17:0	3.73 ± 0.55	ND	-
17:1	0.86 ± 0.05	ND	-
18:0	25.98 ± 2.64	23.51 ± 0.26	0.403
18:1n9	18.65 ± 1.37	14.64 ± 0.28	0.060
18:1n7	1.74 ± 0.15	1.15 ± 0.12	0.039
18:2n6	3.04 ± 0.53	4.86 ± 0.18	0.031
18:4n3	1.06 ± 0.29	ND	-
20:1n9	ND	0.54 ± 0.22	-
20:2n6	ND	2.85 ± 0.05	-
20:3n6	0.56 ± 0.24	1.91 ± 0.03	0.004
20:4n6	2.01 ± 1.16	10.89 ± 0.24	0.001
20:5n3	ND	0.35 ± 0.00	-
21:0	0.81 ± 0.11	ND	-
22:4n6	ND	4.29 ± 0.02	-
22:6n3	1.05 ± 0.96	4.62 ± 0.14	0.021
23:0	ND	1.25 ± 0.01	-
24:1	0.42 ± 0.42	1.73 ± 0.17	0.043
∑SFA	70.52 ± 3.45	49.94 ± 0.29	0.004
∑MUFA	21.76 ± 1.64	20.28 ± 0.57	0.443
∑PUFA	3.60 ± 0.77	9.62 ± 0.20	0.001
∑LCPUFA	4.12 ± 2.08	20.15 ± 0.08	0.001
∑PUFA n3	2.11 ± 0.93	4.98 ± 0.14	0.037
∑PUFA n6	5.61 ± 1.93	24.80 ± 0.42	0.000
Ratio n3/n6	0.35 ± 0.05	0.20 ± 0.01	0.055
Ratio DHA/ARA	0.31 ± 0.20	0.43 ± 0.02	0.610
Ratio ARA/EPA	ND	30.86 ± 0.85	-
Ratio DHA/EPA	ND	13.09 ± 0.31	-

Values represent means ± standard error of the mean (n = 3 animals per treatment). [a,b] Different letters indicate statistical differences between the experimental diets by Tukey's test ($p < 0.05$), the letter a refers to the highest value while the letter b refers to the lowest. ∑SFA, ∑MUFA, ∑PUFA, ∑LC-PUFA, ∑n-3 PUFA, ∑n-6 PUFA are the sum of saturated fatty acids (SFA), monounsaturated (MUFA), polyunsaturated (PUFA), polyunsaturated fatty acids from long chain (LC-PUFA), n3 polyunsaturated (n-3 PUFA) and n6 polyunsaturated (n-6 PUFA), respectively. DHA/ARA is the ratio between the sum of total DHA (docosahexaenoic acid, 22:6n-3) and ARA (arachidonic acid, 20:4n-6); ARA/EPA is the ratio between the sum of total ARA (arachidonic acid, 20:4n-6) and EPA (eicosapentaenoic acid, 20:5n-3); and DHA/EPA is the ratio between the sum of total DHA (docosahexaenoic acid, 22:6n-3) and EPA (eicosapentaenoic acid, 20:5n-3).
